# Supporting US healthcare providers for successful vaccine communication

**DOI:** 10.1186/s12913-023-09348-0

**Published:** 2023-05-02

**Authors:** Amanda J Pierz, Lauren Rauh, Dima Masoud, Alanna Kate Cruz, P. Christopher Palmedo, Scott C Ratzan, Ruth Parker

**Affiliations:** 1 Department of Community Health and Social Sciences, CUNY Graduate School of Public Health and Health Policy, City University of New York, 55 W 125th Street, New York, NY 10027 USA; 2 Division of General Medicine, Emory University School of Medicine, Atlanta, GA USA

**Keywords:** Health care providers, Vaccine trust, Vaccine communication, COVID-19, Patient-provider dyad

## Abstract

**Background:**

While many healthcare providers (HCPs) have navigated patients’ vaccine concerns and questions prior to the rollout of the COVID-19 vaccines, sentiments surrounding the COVID-19 vaccines have presented new and distinct challenges.

**Objective:**

To understand the provider experience of counseling patients about COVID-19 vaccinations, aspects of the pandemic environment that impacted vaccine trust, and communication strategies providers found supportive of patient vaccine education.

**Methods:**

7 focus groups of healthcare providers were conducted and recorded during December 2021 and January 2022, at the height of the Omicron wave in the United States. Recordings were transcribed, and iterative coding and analysis was applied.

**Results:**

44 focus group participants representing 24 US states with the majority (80%) fully vaccinated at the time of data collection. Most participants were doctors (34%) or physician’s assistants and nurse practitioners (34%). The negative impact of COVID-19 misinformation on patient-provider communication at both intrapersonal and interpersonal levels as well as barriers and facilitators to patient vaccine uptake are reported. People or sources that play a role in health communication (“messengers”) and persuasive messages that impact behavior or attitudes towards vaccination (“messages”) are described. Providers expressed frustration in the need to continuously address vaccine misinformation in clinical appointments among patients who remained unvaccinated. Many providers found value in resources that provided up-to-date and evidence-based information as COVID-19 guidelines continued to change. Additionally, providers indicated that patient-facing materials designed to support vaccination education were not frequently available, but they were the most valuable to providers in a changing information environment.

**Conclusions:**

While vaccine decision-making is complex and hinges on diverse factors such as health care access (i.e., convenience, expense) and individual knowledge, providers can play a major role in navigating these factors with their patients. But to strengthen provider vaccine communication and promote vaccine uptake, a comprehensive communication infrastructure must be sustained to support the patient-provider dyad. The findings provide recommendations to maintain an environment that facilitates effective provider-patient communication at the community, organizational and policy levels. There is a need for a unified multisectoral response to reinforce the recommendations in patient settings.

**Supplementary Information:**

The online version contains supplementary material available at 10.1186/s12913-023-09348-0.

## Background

The importance of healthcare provider (HCP) patient communication has been thrust in the spotlight during the COVID-19 pandemic. Facing vaccine questions and concerns about vaccination in patient interactions is not a new challenge for many HCPs working in United States (US) health care settings. A large body of research suggests strategies that US providers can deploy to improve health communication and increase vaccine acceptance. Some recommendations include more time with patients during appointments [1], using motivational interviewing techniques [2], and allowing for honest discussions about vaccine concerns [3]. While evidence has shown that US-based family physicians [4] and pediatricians [5] can have a great role in increasing vaccine acceptance, especially for vaccine-hesitant parents [6–8], this places a heavy burden on the provider to change often strongly held beliefs.

Communication challenges have been exacerbated by the spread of false information on social media and other media outlets. This relentless misinformation coupled with an evolving information landscape submerged the public (including healthcare providers) in an overwhelming *infodemic*. As a result of this swirling information environment, there was an increasing demand on providers to provide up-to-date information to patients, navigate information voids, and combat resulting questions, concerns, and vaccine hesitancy [9]. Throughout the COVID-19 pandemic, the communication resources available to providers were often insufficient to address patient questions [10] in a highly fluctuating and increasingly polarized environment. The distinct challenges presented by COVID-19 vaccine attitudes and misinformation offer many considerations for continued practice, [11] and new solutions [5].

Unique environmental and policy factors in the US that persisted during COVID-19 pandemic and impacted HCP’s ability to provide patient care and education. In the US, controversies over compulsory vaccination have a complicated history, with COVID-19 as a prime example of the tension between protecting the health of the public and safeguarding the civil liberties of American citizens [12, 13]. Many state laws require vaccinations to reduce the rate of vaccine-preventable disease, such as those mandated for children to enter day care or school and federal employees to physically work within government buildings and facilitates [14]. However, US communities with varying levels of suspicion and mistrust of vaccines, persistent health care access inequities, religious exemptions, and predatory disinformation have threatened herd immunity targets for some vaccinations and resistance to vaccination mandates [15, 16]. Cultural aspects of public responses to COVID-19 in the US and increased exposure to misinformation campaigns throughout the pandemic magnified need to improve health communication strategies and strengthen the patient-provider relationship [11]. As a result of these complexities in the US context, vaccine communication can be challenging to address the myriad of concerns.

Due to high levels of institutional and government distrust, provider communication plays a key role in vaccine acceptance. In a report by WHO (2014), patient education during routine care led to the greatest increase in vaccine uptake [17]. Evidence-based education and training are crucial for clinicians to increase vaccine confidence [18], and as seen during the COVID-19 pandemic, are complemented by external factors such as vaccine availability and directing patients to trusted sources [19]. Comprehensive and consistent efforts are especially important for pregnant patients [20] and for Black Americans who consistently have lower rates of flu vaccination [21].

In an emerging body of literature, HCP experiences and perspectives are explored to assess how COVID-19 vaccine attitudes have impacted patient-provider relationships. Using a series of focus groups with HCP across the United States, the aim of this study was to capture and analyze the provider experience of patient counseling for COVID-19 vaccinations and how different aspects of the pandemic environment have impacted vaccine trust. We present our findings from these focus groups and offer recommendations for strategies to support provider health communication broadly.

### Theoretical framework

**Fig. 1:**
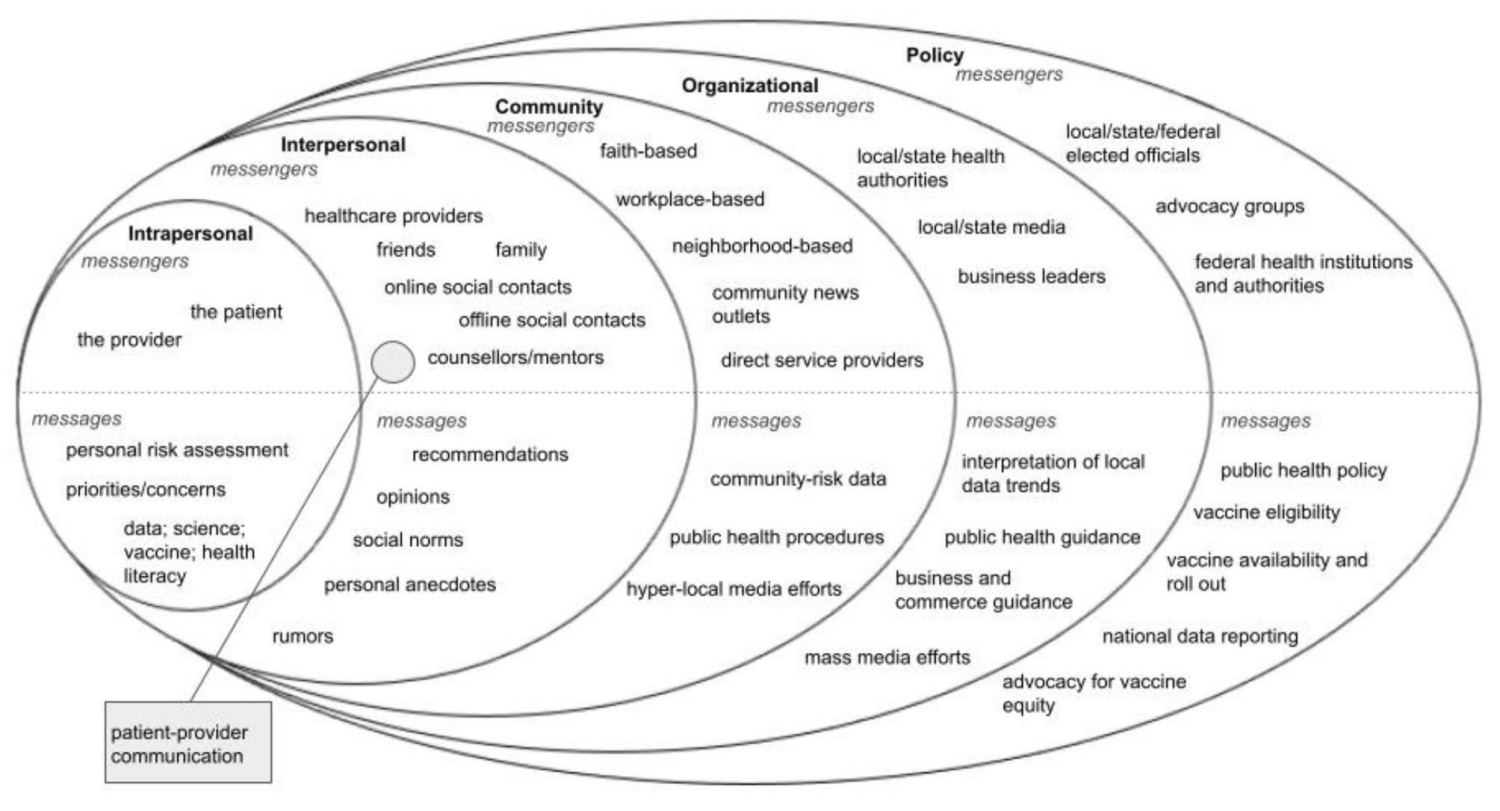
Socio-ecological Model of Vaccine Communication

Vaccine decision-making is influenced by a variety of psychosocial and environmental factors that form a complex ecosystem of factors that facilitate or create barriers to vaccine acceptance [22–24]. Kincaid et al. (2004) conceptualized a model of communication for social and behavior change across embedded sectors within the individual, social networks, community, and societal [25]. While there is a breadth of research describing the strategies healthcare providers can employ to encourage vaccine uptake among their patients, there is little in the way of understanding how to best support healthcare providers at the community, organizational or policy level. We borrow from Kincaid et al. (2004) and the Socioecological Model developed by Bronfenbrenner (1977) to interpose communication influences and modes at each level in the larger communication context, demonstrating support for providers needs to stem from outer levels to encourage individual-level change [25, 26]. We present a suggested Socio-ecological Model of Vaccine Communication in Fig. 1, including common *messengers* and *messages* at each level. Each level of the model illustrates messengers that have an influence within that particular level based on the narrative provider data captured in this study. Additionally, the model provides a selection of various messages observed across each level of vaccine communication.

## Methods

To screen and recruit focus group participants, we collaborated with Alligator Digital, a third-party panel provider to field a survey across the United States from October 19 – November 12, 2021. Alligator Digital conducted the survey with 524 complete opt-in computer-assisted web interviews (CAWI), composed of medical professionals. The panel data were used to support a purposive sampling strategy of eligible healthcare professionals to participate, including doctors, nurses, and other medical professionals. In line with the study’s research questions, the HCP sample was designed to capture and segment the perspectives of healthcare providers most likely to counsel patients on a regular basis. Participants who reported they did not discuss vaccination with patients were ineligible and excluded from focus group discussions (FGDs). HCPs interested in participating in a FGD provided their contact information when surveyed. All FGD recruitment was conducted by the research team via email.

Based on the results from the national survey and input from an advisory group of experts in health communication, health behavior, and vaccine confidence, the following domains were identified as key areas to include in the focus group discussion guide (Appendix A): (1) best practices and strategies to discuss vaccination with patients; (2) preferred and helpful sources of information; (3) impacts of COVID-19 on the work environment; (4) perspectives on the HCP role in combating vaccine hesitancy; and (5) recommendations for supporting vaccine uptake. Through qualitative data collection and informed by our conceived model, data analysis aimed to define actionable items and communication strategies to improve vaccine acceptance among residents of the United States. This study was reviewed by the Internal Review Board at the City University of New York (CUNY) Graduate School of Public Health and Health Policy (SPH) as part of a larger mixed methods project, protocol number 2021-0330-PHHP. Findings from the full study are reported elsewhere [27, 28].

All focus group discussions (FGD) were segmented by profession and vaccination status: two groups of vaccinated physicians, two with vaccinated nurse practitioners (NP), two with vaccinated registered nurses (RN), and physician’s assistants (PA) and one with unvaccinated HCPs of various professions. Focus group size ranged from 4 to 7 participants per session depending on attendance and lasted 60–75 min. FGD were conducted until reaching thematic saturation. All participants were compensated with a $250 online gift card for their participation in the study. Focus groups were conducted by two members of the research team in December 2021 and January 2022. Before participating in the FGD, all recruited HCPs provided verbal informed consent and explicitly provided permission to be audio recorded as approved by the Internal Review Board at CUNY SPH. All groups were conducted and recorded through Zoom and audio files were transcribed for qualitative analysis.

The research team developed an initial codebook based on the interview guide domains and made iterative revisions through a first round of coding [28]. To ensure intercoder reliability, [29] at least two team members were assigned to each transcript to code and develop analytic memos of the transcripts [30]. Thematic analysis embedded within our model of Vaccination Communication of HCP. The research team met regularly to update current codes and discuss analytic approaches through an iterative approach to finalize the relevant themes as presented. Preliminary findings were sent to an advisory council for feedback following a roundtable discussion. The report was subsequently distributed to participants of the study to check for accuracy and ensure that the report reflected their experiences [31]. Six participants replied via email to confirm the report adeptly summarized their perspectives.

## Results

Table 1 describes the demographics and characteristics of the 44 focus group participants. The majority of participants were doctors (34%) and physician’s assistants or nurse practitioners (34%). The majority (80%) were fully vaccinated at the time of data collection. Twenty-four US states were represented and included all regions of the country. The states with the largest representation were Indiana, North Carolina and Texas. Participants were primarily Democratic (41%), white (77%) and female (75%).

**Table 1:** Sociodemographic data from HCP participants

	*Frequency* (n = 44)	*Percentage*
**Provider Type**		
Doctors	15	34%
PAs & NPs	15	34%
RNs	12	27%
Other (Medical Assistant)	1	2%
Pharmacists	1	2%
**COVID-19 Vaccination Status**		
Fully vaccinated	35	80%
Unvaccinated	9	20%
**Political Affiliation**		
Democrat	18	41%
Republican	13	30%
Independent	8	18%
No preference	5	11%
**Race/Ethnicity**		
White, non-Hispanic	34	77%
Black, non-Hispanic	6	14%
Asian	3	7%
Multiple	1	2%
**Gender**		
Female	33	75%
Male	10	23%
Non-binary/gender non-conforming	1	2%

Results at the intrapersonal and interpersonal level demonstrate the impact of COVID-19 misinformation on patient-provider communication and potential messengers and messages that can play a role in either promoting or combating misinformation. Results at the community, organizational and policy levels reveal key sources of information and recommended strategies to create an environment that supports vaccine acceptance. Table 2 summarizes the thematic analysis and provides excerpts from the FGDs for each of the themes identified.

**Table 2:** Qualitative Constructs and Themes

Construct	Theme	Socio ecological Messengers	Illustrative Quote
*COVID-19 misinformation has altered the patient-provider relationship*	Misinformation brought to appointments creates barriers to discuss science-based recommendations; providers face increasingly “dug in” perspectives.	Interpersonal	*There’s a lot of misinformation, even the people who got the vaccine…I can’t in a few minutes visit just prove it or bring out all the data. I’m not even sure if that would help… I have that discussion with [people] that have very firm beliefs…It’s almost impossible to get through.* (Nurse practitioner, Illinois)
			*At this point, it’s almost like they’re dug in, and they would be embarrassed that they’ve* *changed their mind. And I don’t know how to get through to them to let them know that would be good. (Doctor, North Carolina)*
	Some patients believe the information made available to providers is wrong – increasing doubt in unbiased science and medicine	Interpersonal	*They trust us as their physicians. They trust that we’re in good faith trying to give them the best information we can. It’s just that they’ve been somehow misled that the information that we have as medical professionals is incorrect and they feel like… They’ve got access to truth that the medical professionals don’t.* (Doctor, North Carolina)
			*It’s not that they think that I have a hidden agenda and that I’m part of an evil conspiracy, it’s that they think that the doctors are actually misinformed. They think we believe what we’re telling them, we’re just wrong and they have a better source than we do. (Doctor, North Carolina)*
	A paradigm shift for some providers in how they comprehend and communicate new medical information	Intrapersonal	*I’ve had patients share information that they’ve discovered that’s contrary to what we’ve been taught to teach through the years. It’s been sort of paradigm changing for me. To be this old in medicine and to feel like my paradigm has changed. So, it becomes harder to talk to patients when you get different information than what you had your whole life.* (Unvaccinated doctor, Pennsylvania)
			*I’ve definitely taken to heart more concerns and anecdotal stories that patients have brought either from their own vaccine stories or from loved ones. It’s really easy to blow some of these things off and point to studies. Well, you got people flooding in with all these stories that you start taking [them] to heart, especially something that’s been so newly released and not very well studied. You kind of internalize that. It’s hard not to bring that to other patients that you interact with.* (Unvaccinated doctor, Wisconsin)
*Strategies for successful vaccine communication during patient interactions*	Tailoring information and recommendations to the patient’s medical history and concerns	Interpersonal and Intrapersonal	*Knowing what their literacy level governs a lot on the terminology that we use… give them a chance to ask questions, and the pros and the cons that type of thing… You really have to know the background of the people, and that makes a big difference.* (Nurse practitioner, Massachusetts)
	Testimonials from other patients and peers	Interpersonal	*I said it in a conversation… “I just lost a patient on the vent with COVID,“ and the lady went down that day and got the vaccine. So, some of [their choices] are personal.* (Nurse practitioner, Indiana)
			*I asked her if she would go in and speak with a woman who received her first [dose]. They just had a conversation and found out that they went to the same college. They actually exchanged numbers and became friends right there. I thanked her for making her feel comfortable and telling her that you didn’t have any side effects. So, sometimes I use that method.* (Registered nurse, Texas)
	Time, multiple appointments, patience and empathy	Intrapersonal (with organizational barriers due to health care access, appointment-making policies, etc.)	*It’s an ongoing conversation. I tell them [to] think about what we talked about, and we can discuss it again. If you change your mind or if you want to discuss this further, we can always address it out at a later time… so they don’t feel pressured.* (Nurse practitioner, New York)
*Information resources that support provider vaccine communication*	Local and frequent updates	Community, Organizational and Policy	*Our local public health department disseminates our information a couple times a week… and they recap the new CDC guidelines and what the State of Iowa’s Department of Public Health recommends.* (Nurse practitioner, Iowa)
	Resources and materials provided by employers	Organizational	*I work for a huge hospital so the epidemiologist at the hospital would send out daily updates, and they were translating the information that was in the media and on the CDC website. It was very simple to follow, it also included instructions for patients on what to tell them… regarding vaccines, side effects, protocols, and step-by-step approach on how to navigate the world of COVID. I found that very helpful.* (Nurse practitioner, Utah)
	An increased need for patient-facing materials to navigate new COVID-19 information	Community and Organizational	*I would like to really have a resource to provide to families and patients…in a format that’s really easy to follow… A lot of us don’t have a ton of time to explain to families, but to give them an opportunity to…do a little bit of research on their own without maybe digging through the CDC website.* (Nurse practitioner, Texas)
*Environmental and policy level recommendations to support vaccine acceptance outside the clinical setting*	Removing financial barriers to vaccination	Policy	*…I am in a pediatric private practice…we do have a lot of parents asking the same thing, “is it covered by insurance?”…* (Nurse practitioner, Indiana)
	A need for a more centralized and unified response to COVID-19 vaccinations	Organizational and Policy	*Other countries are doing a much - I don’t want to say a better - but a different job of [communicating] this is just for the good of the population, and… try to get people to get more into that community mentality.* (Nurse practitioner, Indiana)
	Multiple outlets for information and vaccination	Community, Organizational and Policy	*We’ve gone to COVID clinics… at different churches. So, I think as far as culture is concerned some people they really look at their priests, their minister, their pastor, for guidance… If you could get him… on board to endorse the vaccines, I think you will get more participants. I really do.* (Registered nurse, Texas)
			*Who speaks to the different groups? Maybe our current administration doesn’t speak to everybody but somebody speaks to everybody. Somebody reaches. Every person out there has somebody they respect or that they know and trust. And, maybe we need to move away from the national speakers that everybody’s seen on TV all the time. Maybe regionally, they need to look for people of different race, color, interests, backgrounds and find somebody to speak locally or regionally to those that are hesitant. I mean, that’s the only thing I can think of is to better reach individuals.* (Doctor, Nebraska)

### Intrapersonal and interpersonal vaccine communication

#### COVID-19 misinformation has altered the patient-provider relationship

Overall, the focus group participants largely viewed their role as providing a source of scientific information and patient education during appointments. They saw themselves as trusted messengers for their patients, community, friends and family, but were quick to note communicating this information became more challenging during the COVID-19 pandemic. On the topic of COVID-19 vaccine hesitancy and refusal, most providers felt they had offered sufficient patient education and intervention in the year since the COVID-19 vaccine became widely available and that most unvaccinated individuals were no longer open to being counseled.

Providers expressed that, for the first time, some of their patients had doubts about their clinical guidance, believing that they were influenced by pharmaceutical or other institutional forces. While most providers did not face direct accusations of purposely misleading patients (especially those with long-standing relationships with their patients), providers faced patients who expressed distrust of the accuracy of information they offered.

A group of unvaccinated and/or “late adopting” providers (defined as being vaccinated after November 2021) indicated that they experienced a shift in perception of their own role in vaccine promotion. They expressed distrust stemming from their belief that vaccine mandates were implemented without comprehensive scientific evidence to support them, such as a lack of consideration for natural immunity in vaccine policy development. Importantly, these providers shared many of their patients’ COVID-19 vaccine concerns and reported that information provided by patients led them to question some key aspects of their medical training.

#### Provider’s strategies for vaccine communication during patient interactions

The providers offered several strategies for promoting vaccine acceptance among patients. The most common strategy was to tailor information to each patient’s medical history and concerns related to the COVID-19 vaccine and to avoid generic guidance. In their view, this approach facilitated provider trust and mitigated any institutional mistrust. This communication strategy was echoed as effective and meaningful in subsequent in-depth interviews and focus groups with patients in the parent study reported elsewhere [21].

A few providers touted *“scare tactics”* that appeal to patient fear, stating that patients have responded to other vaccine recommendations which cautioned of severe disease outcomes. One provider suggested this is an underused patient education tactic for the COVID-19 vaccine, citing the success of anti-smoking campaigns that highlighted severely impacted former smokers with chronic illness and disability. Providers also found that testimonials from recent adopters had an impact on their patients. Providers described the sharing of personal anecdotes, family stories, and introductions to other recently vaccinated patients as helpful for individuals still uncertain of their vaccination decision. Details about the unknown and prolonged effects of long-COVID would be an example of this communication strategy.

When discussing successful strategies for patient communication, many providers acknowledged that addressing vaccine hesitancy often takes multiple appointments with the same patient, and adequate appointment time - both circumstances that many patients and providers cannot independently facilitate or control. Those who saw patients on a regular basis due to the type of care they provided (i.e., maternal, and prenatal health care; care for chronic conditions) noted that the ability to have multiple touchpoints with the same patient facilitated a trusting dialogue around medical recommendations, including vaccination.

### Community, organizational and policy messengers

#### Information sources that support provider vaccine communication

Many providers discussed the pressure to stay up to date in an evolving information environment, especially during the first year of the pandemic. They had mixed opinions on whether they had adequate resources to answer patient questions about the COVID-19 vaccination but generally agreed about their main sources of vaccine information during the pandemic. Participants cited the Centers for Disease Control and Prevention (CDC) and professional organizations like the American College of Obstetricians and Gynecologists (ACOG) as helpful. Other helpful resources included local and state health departments whose regular updates mitigated pressure on healthcare providers to stay up to date.

Several providers also indicated workplace communication digests and regular team meetings led by department heads as the most helpful resources to stay current on COVID-19 information. Some of these communications from employers also included patient-facing resources, which many providers reported as necessary to facilitate conversations with new information.

#### Provider’s recommendations to support vaccine acceptance outside the clinical setting

Providers described the challenge of addressing COVID-19 vaccine questions and concerns in an environment that often left them unsupported to reduce barriers to vaccination. This discussion led to some clear guidance for policy and institutional practices to address providers’ barriers to vaccine counseling. Firstly, many providers recommended all vaccinations be provided free of charge to the patient. Providers highlight patient financial concerns surrounding the COVID-19 as well as previous vaccinations. Despite the national provision that vaccines be available free of charge, there continued to be confusion among patients about the financial cost of vaccination. This has implications for both communication strategies and ready access to vaccines.

Some providers suggested continuing to offer vaccinations outside of the medical office or hospital environments (i.e., at mobile units, or pharmacies) to prevent cold supply chain challenges and other barriers in small doctors’ offices. Many providers hoped that the lessons learned during the COVID-19 vaccine rollout will inform future vaccination availability.

Providers indicated a strong need for a more centralized, unified vaccine communication response from regional and federal agencies to address the ongoing challenges that they face addressing oft-conflicting vaccine messages from health officials, and government representatives. While they recommend the policies and messaging come from a centralized effort, there is additional importance of engaging local messengers. They underscored the need for local, diverse and neutral messengers from trusted community leaders to combat further politicization and polarization. Some acknowledged this could involve collaboration between other sectors that may not be traditionally involved in public health campaigns (e.g., community leaders, faith leaders).

## Discussion

We discuss our findings in the context of this model, focusing specifically on solutions to mitigate the negative impacts of misinformation, or evolving or confusing information on the patient-provider relationship and suggestions to create a stronger communication infrastructure that anchors patient-provider relationships. The results of this study highlight the impact of the confusing and often chaotic information environment surrounding COVID-19 and COVID-19 vaccination on patient-provider communication and demonstrate the strain caused when HCPs are not supported to respond adequately to patient concerns during clinical interactions. The impacts of misinformation specifically on the patient-provider dyad during the pandemic may be indicative of a new chapter of vaccine sentiments influencing how HCPs approach conversations about vaccination [32] While HCPs can play an important interpersonal role in providing consistent and empathetic messaging for their patients, policies and procedures must strengthen organizational and community communication channels to better provide consistent evidence-based health information. Our findings are situated in the socioecological model to show that HCPs counsel patients within a complex communication environment and can be helped or hindered by community, organizational, and policy factors. Viewing patient-provider communication this way demonstrates, for example, that HCPs cannot be the sole combatant to pervasive and predatory misinformation as the public is exposed through various means.

One of the most salient findings from our analysis was the provider recommendation for tailored interpersonal communication strategies that “meet patients where they are at.” This includes the empathetic recognition of patient questions and concerns but also the personalization of their advice to each patient’s medical needs – key strategies echoed in motivational interviewing and other tailored approaches [33, 34]. Providers generally agreed that vaccine acceptance requires an iterative and multi-phased process for many patients [17, 35]. The inclusion of anecdotes and personal perspectives has been demonstrated to be particularly effective in combating anti-vaccine misinformation [36].

Our findings echo current COVID-19 communication literature describing HCPs as the most effective messengers to present tailored messaging to their patients [37–39]. However, to ensure the sustainability of such approaches on a community-level and prevent provider burnout, other resources are needed to support broader efforts to address vaccine trust.

To provide the best evidence-based communication approaches, providers require support to navigate changing medical advice while fielding patient’s questions and concerns. For example, our participants agreed that guidance condensed into digests at a regular cadence (i.e., weekly) was the easiest way to consume new information. Digests were most helpful when they came from local organizational-level messengers like a regional health department or an employer. Having hyper-local data snapshots, news and guidance mitigated the pressures felt by providers to be consistently up-to-date and helped them tailor their information to their patients given the regional nature of the pandemic experience. Unlike many web pages or e-newsletters, these channels should flow two ways to include feedback from the providers on the use and usefulness of the resources provided. While we found communication resources are most effective and impactful when tailored at the community-level, national policy and advocacy must support the collection and dissemination of up-to-date, evidence-based information that all can access and use.

Furthermore, our findings indicate that providers seek resources to combat misinformation and overcome entrenched myths and misconceptions beyond currently available educational materials and resources. Bonnevie et al. (2021) have called for the development of partnerships to monitor and track sources of vaccine misinformation and responses to such campaigns through existing monitoring systems in our health infrastructure [40]. Based on our findings, we recommend that communication and response infrastructure is set up between private organizations tracking and combating misinformation, clinical facilities providing patient education, and government actors with the resources and capital to ensure the sustainability of the collaborative before the next pandemic.

The decline in government subsidies for COVID-19 vaccines and testing may impact the acceptability and uptake of vaccination and other mitigation measures [41]. Our focus group participants believed removing financial barriers to vaccination would increase patient uptake, citing that prohibitive cost to patients would thwart any effective communication efforts. Affordable vaccinations and availability at convenient locations removes logistical barriers for vaccine willing patients. As a policy-level intervention to encourage vaccine acceptance, free or low-cost vaccination in the US must be sustained, regardless of income, insurance, or legal status.

The polarized and politicized information environment during the COVID-19 pandemic had a significant impact on vaccine trust and literacy [42–46]. Our participants agreed the lack of a unified response from different US federal, state, and local agencies greatly contributed to community fragmentation over COVID-19 preventative measures, including vaccination. Future efforts should be made to ensure a coordinated and unified policy-level response to limit regional and community dissension and enhance public trust and the adoption of public health measures in future emergencies.

While participants spanned the United States, the limited sample size prevents generalized conclusions or assessments of correlations in our results. Although attempts were made to recruit for racial and ethnic diversity, the final sample of participants was largely white and concentrated on the coastal regions of the US. These focus groups offer a preliminary understanding of barriers and facilitators HCPs face when promoting vaccine acceptance. This study highlights the need for further research on perspectives of vaccine trust and acceptance from marginalized and rural populations.

## Conclusion

HCPs faced various unique challenges throughout the COVID-19 pandemic, including unprecedented volumes of mis- and disinformation, often rendering pre-pandemic strategies to tackle vaccine hesitancy ineffective. There is a need to recognize provider perspectives in the creation of vaccine communication programs to mitigate HCP challenges and provide sufficient, up-to-date data to address patient concerns. Most important, HCPs require the support of policies and a communication infrastructure that builds patient trust in health care institutions and the science behind vaccination.

## Electronic supplementary material

Below is the link to the electronic supplementary material.

Supplementary Material 1

## Data Availability

The datasets during and/or analyzed during the current study available from the corresponding author on reasonable request.

## List of abbreviations

ACOG: American College of Obstetricians and Gynecologists
CAWI: computer-assisted web interviews
CDC: Centers for Disease Control and Prevention
COVID-19: Coronavirus
CUNY: City University of New York
FDG: focus group discussions
HCP: health care providers
NP: nurse practitioners
PA: physician’s assistants
RN: registered nurse
SPH: School of Public Health
WHO: World Health Organization
