# Supplementary material for: Supporting US healthcare providers for successful vaccine communication
Source: BMC Health Serv Res. 2023 May 2;23:423. doi: 10.1186/s12913-023-09348-0 (PMC10152412; doi:10.1186/s12913-023-09348-0)
Supplement: Supplementary file 1 — Supplementary Material 1 [file 12913_2023_9348_MOESM1_ESM.docx]

**Appendix A**. Focus Group Discussion Guide for Health Care Workers

Research goal: Aim to understand provider attitudes toward discussing adult vaccination with patients and the resources they need (or have used) to help build a climate of acceptance for, and uptake of, adult vaccinations, including COVID-19 vaccines.

Welcome: Thank you for taking the time to participate in this study. My name is Amanda Pierz, I am a researcher at the City University of New York Graduate School of Public Health and Health Policy. I’m hoping today to speak with you to better understand your experiences counselling patients about adult vaccination and your own feelings about this role. This focus group is part of a study exploring the role of trust in vaccine decision making. Please take a moment to change your name to either your real name or whatever you would like to be called. I may need to use those names to call on you occasionally.

Before we dive in, let’s start with some ground rules to make sure everyone feels comfortable and respected. There are no right or wrong answers. The only right answer is your honest answer. We need to hear your thoughts and opinions! You can say you disagree with someone but please respect views other than your own. If you ever feel uncomfortable answering a question, you can always choose to skip it.

We will be audio recording our discussion today, so we can transcribe it for review by the research team. Please know that when we report our findings, your name will never be associated with anything you say or the fact that you attended this focus group. That’s part of our commitment to confidentiality. On your part, please keep what you hear in the discussion to yourselves, to protect and respect each other’s opinions and feelings. I do also want to encourage you to use the chat, I will try my best to monitor it, but it is a good way to make sure we gather all views and opinions in our short time together.

Today’s focus group will last about an hour. Do you have any questions before we start? [Wait for questions]. I am going to begin the recording.

This is the Vaccine Trust Study, I am Amanda, and this is focus group# 4 for HCW and it is 27 January 2022.

Let’s start by introducing yourself by stating your name and what kind of clinical setting you work in and where you are located in the country. As I said, your name will never be associated with anything you say today but if you still want to use a pseudonym when you introduce yourself that’s OK too!

**Introductory Questions:**

*If people bring up Covid in their intros start here:*

1. So, we are here to discuss counselling adult patients about vaccination generally, but since we are two years into this pandemic and one year into the vaccine roll out, I’m wondering if we could start recalling some of these interactions from this past year. When patients ask you about Covid-19 vaccines, what kind of questions and concerns have come up?

*If Covid does not come up in intros, start here:*

1. I know this may be hard but let’s think back to the world of adult vaccination before COVID-19 (pause). Back then, when your patients discussed adult vaccination, what kinds of questions and concerns came up?
   1. Does anyone have an example of this type of conversation? *Probe, if necessary:*
      1. At what kind of appointments or in what interactions would these discussions come up?
   2. How is this different with Covid-19 vaccines?
2. What kinds of information sources have you found helpful in your conversations about Covid-19 vaccination with patients? I’m talking about websites, pamphlets, other medical professionals, really anything that helped you.
   1. Are there items that were helpful to you in the past, but are no longer?

**Transition Questions:** *(these are only necessary if this info has not already been offered)*

1. What have the attitudes been like toward Covid-19 vaccination in your medical community?
   1. That kinds of things have you seen or heard?
   2. How have these attitudes impacted your consults and interactions?
   3. *If necessary,* can anyone share examples of providers, physicians or otherwise, who were wary of, or opposed to, Covid vaccination?

**Key Questions**:

1. What barriers have you faced to introducing Covid-19 vaccine discussions during patient interactions?
   1. What do you think would help you overcome those barriers? In other words, what would help you have more meaningful and effective conversations with patients about vaccination?
      1. *Possible probes if necessary:* Any education tools or resources?
      2. Changes in the work environment or scheduling structure?
      3. *If this info has not already been covered:* Are there issues that would need to be addressed to allow you sufficient time to counsel patients about a COVID-19 vaccine?
   2. *If discomfort is mentioned,* what will help you feel more comfortable to have these interactions?
2. How do patients express their hesitancy or concerns around vaccination? Has Covid-19 been different? In what ways?
   1. Does anyone have an example of a patient who was vaccine confident but isn’t anymore? What about COVID-19?
   2. Do you think those hesitant patients do not trust the information you give them? Why, why not?
3. What have you found to be the best practices when you are helping patients decide if vaccines are right for them?
   1. Can you share some examples from before the pandemic? Maybe with the flu vaccine or shingles?
   2. Can you share some examples about the Covid-19 vaccine specifically?
4. Thinking about COVID-19 vaccines, are there any concerns people raise that resonate with you? That you sort of agree with? That you sympathize with?
5. *If necessary:* Do you or did you have any personal concerns about the COVID-19 vaccines? If so, could you explain them?
   1. How did you go about seeking answers to these concerns? Did you find what you needed to feel trusting of the COVID-19 vaccine?

***The health care professional role***

1. What do you think about the public expectation that you can and are able to discuss vaccination with patients?
   1. Sometimes health care providers are called “trusted messengers,” what do you think about this designation?
   2. Do you see yourself as a “trusted messenger” of health information?
      1. Why or why not? Please share an interaction with a patient that illustrates this.
2. Do you think there’s pressure placed on physicians to solve the vaccine hesitancy issue?
   1. Is this a role you feel comfortable taking on?
3. If a patient appears to have low awareness or misconceptions about adult vaccines, such as seasonal flu shots, Hepatitis B, Shingles, how do you counsel them? For example, someone might have what’s called “low health literacy” around vaccine effectiveness and safety. Can you share some examples of a typical conversation?

**Ending/Wrap-up Questions:**

1. OK those are the specific questions we wanted to cover. Let me conclude by asking you this: What can we do as a health care and public health community, to improve acceptance of adult vaccines, including Covid vaccines?
2. Is there anything we didn’t touch upon that would be important in order to better understand this topic?

Thank you [end recording]
